# The Dual Nature of Cellular Senescence: From Aging Signature to Regenerative Catalyst

**DOI:** 10.34133/research.0830

**Published:** 2025-08-05

**Authors:** Peng Li, Yi Yang, Xiang Qin, Juan Liao

**Affiliations:** ^1^ Department of Anesthesiology, Sichuan Provincial People’s Hospital, School of Medicine, University of Electronic Science and Technology of China, Chengdu, China.; ^2^ Department of Stomatology, Sichuan Provincial People’s Hospital, School of Medicine, University of Electronic Science and Technology of China, Chengdu, China.

## Abstract

This perspective critically examines the paradigm-shifting findings regarding cellular senescence’s dual role in tissue biology, particularly focusing on its unexpected regenerative potential in hair growth. While cellular senescence has traditionally been viewed as a detrimental process associated with aging and tissue dysfunction, research has revealed its surprising beneficial effects on tissue regeneration. We analyze the groundbreaking discovery that senescent melanocytes can stimulate hair follicle stem cells through the osteopontin–CD44 signaling pathway, challenging the conventional understanding of senescence. This perspective also evaluates the implications of this finding for both basic research and therapeutic applications, suggesting that cellular senescence represents a complex, context-dependent phenomenon rather than a uniformly detrimental process. We discuss how this new perspective necessitates a more nuanced approach to senescence-targeted therapies and opens novel therapeutic possibilities for hair loss treatment. This analysis underscores the importance of understanding senescent cell heterogeneity and their diverse functions in tissue homeostasis, which could lead to more precise therapeutic strategies in regenerative medicine.

## The Traditional View of Cellular Senescence

The long-standing paradigm indicates that cellular senescence represents a primarily detrimental process in tissue biology; however, it was challenged by a research published in *Nature*. Wang and colleagues [1] reveal an unexpected role for senescent melanocytes that challenges the traditional view of cellular senescence as primarily detrimental. Their research demonstrates that clusters of senescent melanocytes can potently stimulate hair follicle stem cells (HFSCs), driving robust hair growth through the osteopontin (OPN)–CD44 signaling pathway. Cellular senescence has traditionally been viewed as a state of permanent cell cycle arrest that contributes to aging [2–4]. When cells become senescent, they stop dividing but remain metabolically active, developing a senescence-associated secretory phenotype (SASP) that includes inflammatory cytokines, chemokines, growth factors, and proteases that can substantially affect surrounding tissues [5]. Conventional understanding suggests that accumulated senescent cells progressively impair tissue function through chronic inflammation and altered tissue microenvironments [6], driving the development of senolytic therapies, which aimed at selectively eliminating these cells [7–9]. This suggests that senolytics could act as a kind of drug that could potentially treat various age-related diseases by clearing out these problematic cells. However, senescent cells also play important roles during embryo development, wound healing, and tissue repair, suggesting different functions in different situations [10,11]. In normal tissues, stem cell quiescence is tightly regulated by the surrounding microenvironment, with proper niche signaling maintaining stem cells in a quiescent state until activated for tissue maintenance or repair. Disruption of this delicate balance can lead to stem cell exhaustion through excessive proliferation or regenerative disorders [12–14]. Understanding the factors that regulate this balance represents a crucial frontier in regenerative medicine, particularly in hair follicles where cyclic regeneration requires precise coordination of quiescence and activation signals.

## The Curious Phenomenon of Hairy Nevi

Wang and colleagues explored the intriguing clinical observation that melanocytic nevi, commonly known as moles, often display unusually abundant and robust hair growth. This phenomenon suggests a localized hyperactivation of HFSCs within the nevus regions. Clinically, this manifests as hair shafts that are thicker, longer, and more densely packed in the areas of skin bearing nevi compared to adjacent normal skin. To investigate the underlying mechanisms, the researchers employed 2 genetically engineered mouse models that recapitulate human melanocytic nevi: the Tyr-Neuroblastoma RAS viral oncogene homolog (NRAS)^Q61K^ mouse model, which mimics congenital nevi through the expression of an oncogenic NRAS mutation in melanocytes, and the inducible Tyr-CreER^T2^;B-Raf proto-oncogene, serine/threonine kinase (BRAF)^V600E^ model, representing acquired nevi by conditional activation of the oncogenic BRAF mutation. Each model was tested with adequate sample sizes across multiple independent experiments to ensure robust statistical analysis. These models specifically target melanocytes derived from the neural crest lineage, enabling precise oncogene expression and subsequent induction of cellular senescence within these cells. Mutations in NRAS and BRAF are well-documented drivers in human melanocytic nevi, making these models highly relevant for studying nevus biology and pathology. In both mouse models, the expression of oncogenes leads to oncogene-induced senescence (OIS) of melanocytes, resulting in the accumulation of senescent melanocyte clusters within the dermis adjacent to hair follicles. Notably, these senescent melanocytes are nonproliferative, characterized by markers such as p15 and γH2AX, and are located outside the hair follicle epithelium rather than inside it.

The researchers observed a striking disruption of the normal hair follicle growth cycle in these mice. Typically, hair follicles undergo a synchronized cycle transitioning through growth (anagen), regression (catagen), and resting (telogen) phases [15–18]. However, in nevus-bearing mice, this coordination was lost, and hair follicles exhibited uncoordinated yet hyperactive growth, with numerous ectopic anagen follicles present at any given time. This hyperactivation of HFSCs leads to the excessive hair growth characteristic of hairy nevi. Further experiments demonstrated that the presence of senescent melanocytes, rather than melanogenesis itself, is critical for this phenotype. For example, albino Tyr-Nras^Q61K^ mice lacking melanin still exhibited enhanced hair growth, confirming that the senescent state of melanocytes drives the effect. Additionally, transplantation of senescent melanocytes into normal skin was sufficient to induce new hair growth, whereas normal melanocytes did not have this effect. This work reveals that dermal clusters of senescent melanocytes in nevi secrete factors that potently stimulate HFSCs to exit quiescence and enter active growth phases, thereby explaining the curious clinical phenomenon of hairy nevi. This finding challenges the traditional view of senescent cells solely as detrimental and highlights their role as active signaling hubs capable of modulating tissue regeneration.

## OPN: The Critical Orchestrator of Senescence-Driven Hair Regeneration

Comprehensive transcriptomic profiling of melanocytes isolated from nevi revealed a distinctive secretory profile, or secretome, enriched with numerous signaling molecules that are substantially up-regulated compared to normal melanocytes. Among 27 markedly increased factors, secreted phosphoprotein 1 (Spp1), the gene encoding OPN, stood out as the dominant candidate mediating the effects of senescent melanocytes on hair follicle biology. The senescence-associated gene signature also included elevated expression of modulators of the WNT signaling pathway such as Frzb, Wif1, and Wisp1, components of the bone morphogenetic protein (BMP) pathway including Bmp4 and Fstl1, as well as insulin-like growth factor (IGF) regulatory proteins and other potent signaling molecules [19–22]. These findings highlight the complex and multifaceted nature of the senescent melanocyte secretome and underscore the importance of WNT and related pathways in regulating HFSC activation and hair growth, representing a key direction for future research.

OPN is a multifunctional glycoprotein extensively studied for its roles in inflammation, immune regulation, and cell survival pathways [23–26]. It exerts its biological effects by interacting with multiple cell surface receptors, notably various integrins and the receptor CD44, thereby activating diverse intracellular signaling cascades across different tissue types [27]. While OPN’s functions have been well characterized in bone remodeling, tumor progression, and inflammatory diseases [28–31], its role in hair follicle biology had remained unexplored prior to this study. The investigators conclusively demonstrated that OPN is the causal mediator responsible for the enhanced hair growth driven by senescent melanocytes in nevi. Purified recombinant OPN protein into wild-type mice induced robust activation of hair follicles, triggering their transition into the growth phase (anagen). Conversely, genetic ablation of Spp1 in nevus-bearing mice completely abolished the ectopic hair growth phenotype, confirming the necessity of OPN in this process. Furthermore, melanocyte-specific deletion of Spp1 recapitulated these results, providing compelling evidence that OPN secretion from senescent nevus melanocytes acts through specialized paracrine signaling to directly stimulate HFSC activation and follicular regeneration.

This discovery has important translational implications. Current U.S. Food and Drug Administration-approved treatments for pattern hair loss, such as minoxidil and finasteride, often exhibit limited efficacy and can cause undesirable side effects [32]. The identification of OPN as a potent endogenous inducer of hair growth opens promising new avenues for therapeutic development aimed at stimulating hair regeneration more effectively and safely. This finding challenges the conventional view of senescent cells solely as detrimental and positions their secretome, particularly OPN, as a valuable target for regenerative medicine and hair loss therapies.

## Mechanisms of HFSC Activation

Mechanistically, senescent melanocytes fundamentally reprogram HFSC function by reshaping their local microenvironment and directly altering the molecular state of these stem cells [33,34]. Transcriptomic analyses of HFSCs isolated from nevus-bearing mice reveal extensive gene expression changes, most notably a pronounced down-regulation of genes that maintain stem cell quiescence, such as key cell cycle inhibitors, alongside robust up-regulation of genes that drive cell cycle progression, migration, and WNT signaling pathways—hallmarks of an activated, regenerative state. Single-cell RNA sequencing further demonstrates that the group of quiescent, telogen-phase HFSCs, which are typically abundant in normal skin, is entirely absent in nevus-bearing mice. Instead, these are replaced by HFSCs, which exhibit a distinct activation signature and marked by elevated expression of proliferation and differentiation markers. Central to this reprogramming is the OPN–CD44 signaling axis: Senescent melanocytes secrete high levels of OPN, a multifunctional glycoprotein that binds to its primary receptor CD44 on HFSCs [35]. This interaction is both necessary and sufficient for the observed hyperactivation of hair growth, as targeted genetic ablation of CD44 in HFSCs completely abrogates the enhanced hair regeneration induced by nevus melanocytes, despite the continued presence of senescent cells and OPN in the microenvironment. These findings compellingly establish that senescent melanocytes, through their specialized secretome and niche-like properties, acquire the capacity to potently regulate stem cell behavior via a defined molecular pathway, overriding the normal inhibitory signals that maintain stem cell dormancy. This niche remodeling not only elucidates the mechanism behind the excessive hair growth seen in hairy nevi but also highlights the broader clinical potential of targeting the stem cell niche for therapeutic hair regeneration, suggesting that controlled modulation of the microenvironment could be harnessed to promote tissue renewal in contexts of hair loss or impaired regeneration.

## Future Directions and Broader Implications

This research unveils several compelling avenues for future investigation both in basic science and clinical applications. A particularly intriguing aspect is the differential response of melanocytes to oncogenic stimulation based on their anatomical location. Hair follicle melanocytes demonstrate resistance to OIS, while dermal melanocytes readily undergo this process. This observation suggests the existence of powerful microenvironmental factors within follicles that can counteract senescence pathways. Understanding these protective mechanisms could provide valuable insights for developing targeted anti-aging interventions. The potential existence of similar senescence-driven regenerative mechanisms in other rapidly renewing tissues remains an open question. Investigation of these interactions across diverse organ systems could reveal conserved signaling networks that govern regenerative responses, potentially leading to broader therapeutic applications beyond hair regeneration. As illustrated in Figure, recent breakthroughs in understanding senescent cells have profound implications for hair growth regulation. The study fundamentally shifts our understanding of how senescent cells influence hair follicle dynamics and regeneration processes (Figure). Building on these principal findings, several pivotal advances in clinical applications have emerged that substantially expand therapeutic horizons. The identification and characterization of critical signaling pathways through which senescent cells contribute to HFSC activation and proliferation (Figure) reveals novel molecular targets for intervention. The identification and characterization of critical signaling pathways, particularly the OPN–CD44 axis, opens new possibilities for therapeutic intervention. These pathways offer specific molecular targets for drug development and treatment optimization. The development of innovative OPN-mediated therapeutic approaches represents an important advancement, as leveraging the natural secretory profile of senescent cells could provide more effective and physiological means of stimulating follicular regeneration (Figure). Furthermore, strategic remodeling of the hair follicle microenvironment (Figure) shows promise for improving transplantation success rates and promoting sustainable hair regeneration. This approach addresses current limitations in hair restoration therapies by enhancing graft survival and integration. This research fundamentally reinforces the concept that cellular senescence represents a diverse spectrum of phenotypes rather than a singular state. This understanding necessitates comprehensive characterization of senescent cell heterogeneity for developing precision approaches that selectively modulate senescent cell activities. Future research directions should focus on developing more methods to identify and manipulate specific senescent cells, which could lead to more effective and targeted therapeutic strategies in regenerative medicine and the age-related disorders. These advances hold promise not only for promoting tissue regeneration but also for preventing aging-related pathologies and enhancing therapeutic outcomes across multiple disease states.

**Figure.**
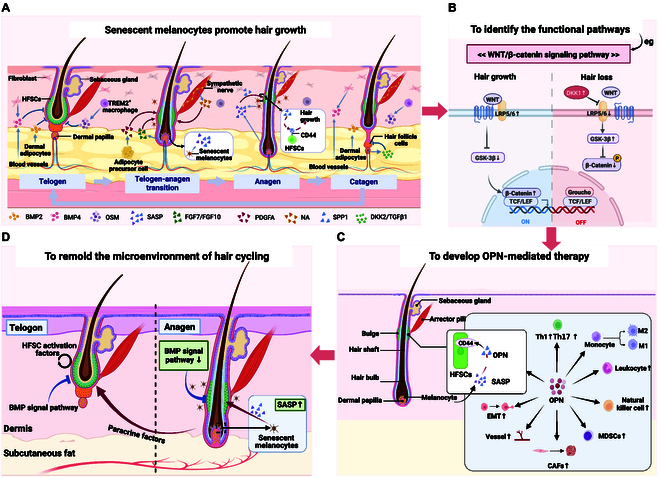
Recent breakthroughs in understanding senescent cells and their potential therapeutic applications in hair growth regulation. (A) Hair follicle cycle progression showing the dynamic interaction between senescent melanocytes and hair growth. The diagram illustrates 4 key stages: telogen (resting phase), telogen–anagen transition, anagen (active growth phase), and catagen (regression phase). Senescent melanocytes accumulate in the dermal region and secrete various factors including BMP2, BMP4, SPP1, fibroblast growth factor 7 (FGF7)/FGF10, and platelet-derived growth factor A (PDGFA). These factors interact with hair follicle stem cells (HFSCs) through specific receptors, particularly CD44, to promote hair growth activation. (B) Molecular signaling pathway analysis revealing the complex interplay of WNT/β-catenin signaling and associated pathways. The diagram details the interaction network involving key molecules such as WNT, glycogen synthase kinase 3β (GSK-3β), and β-catenin, alongside the regulation of downstream targets. This pathway mapping provides crucial insights into potential therapeutic intervention points. (C) Detailed illustration of the OPN-mediated therapeutic mechanism. This shows how OPN secreted by senescent melanocytes interacts with various cell types including HFSCs, immune cells (Th1, Th17, M1/M2 macrophages, and natural killer cells), and other components of the hair follicle microenvironment. The complex signaling network demonstrates OPN’s central role in coordinating cellular responses for hair regeneration.(D) Remodeling of the hair follicle microenvironment, emphasizing the spatial organization of key components. The illustration shows how BMP signaling pathway modulation and paracrine factors influence HFSC activation in the dermis and subcutaneous fat layers. This strategic remodeling approach aims to optimize the local environment for sustained hair growth and improved transplantation outcomes.
